# Efficacy and Safety of Oral Euphorbia prostrata Tablet and Topical Cream in the Management of Hemorrhoids During Pregnancy: Results From a Prospective Multicenter Study

**DOI:** 10.7759/cureus.54152

**Published:** 2024-02-13

**Authors:** Ashwin Porwal, Paresh Gandhi, Nameeta Mokashi-Bhalerao, Nilesh Borkar, Kunal Khobragade

**Affiliations:** 1 Surgery, Healing Hands Clinic, Pune, IND; 2 Obstetrics, Gynaecology and Reproductive Medicine, Cloudnine Hospital, Pune, IND; 3 Medical Affairs, Mankind Pharma Ltd., Navi Mumbai, IND

**Keywords:** post-delivery, pain at defecation, per-rectal bleeding, hemorrhoids during pregnancy, euphorbia prostrata

## Abstract

This is a non-randomized, open-label, prospective single-arm interventional multicentric study conducted between 2021 and 2022 at three different centers situated in Pune, India. It was conducted to evaluate the efficacy and safety of the Sitcom^®^ tablet (*Euphorbia prostrata *100 mg) once daily and Sitcom^®^ cream (*Euphorbia prostrata* 1%w/w) for 14 days in hemorrhoids during pregnancy. The endpoints were to assess hemorrhoidal symptoms relief during the follow-up periods (one, two, four, and eight weeks and 30 days postpartum), relapse of symptoms, improvement in the disease condition at week two and 30 days postpartum, and adverse events. A total of 100 patients (mean age 24.1 years) were included; the majority (71.0%) had mild per-rectal bleeding, 69.0% with mild itching and 46.0% with moderate pain during defecation. The mean score of per-rectal bleeding and pain at defecation showed a significant reduction at each visit (86.6% and 49.3% (two weeks), 95.3% and 59.9% (four weeks), and 100% and 77.6% (eight weeks)). The mean pain score at defecation, itching, exudates, and swelling showed a significant reduction of 77.6%, 96.9%, 100%, and 84.5% at eight weeks (*p*<0.001). After two weeks and post postpartum follow-up, >90% and 100% good to excellent overall improvement in the disease condition were noted, respectively. No adverse events in the mother or newborn were noted. This pivotal study underscores the potential of a combination therapy with *Euphorbia prostrate *100 mg tablet and cream 1% as a potential solution for managing the distressing burden of hemorrhoids in pregnant women. Furthermore, these observations will empower clinicians in the judicious selection of the most suitable course of action for hemorrhoid management during pregnancy.

## Introduction

The development of hemorrhoids occurs in around 40% of women during pregnancy and post-delivery. The impact of this condition resonates deep within, inflicting not only physical discomfort but also taking an emotional toll and deteriorates their quality of life [1,2]. The suffering and strain experienced by women struggling with hemorrhoids during pregnancy is a truly distressing ordeal. Multiple physical complaints or intense discomfort due to relentless pain, swelling, and itching constantly highlight extraordinary burden carried by these women during their motherhood journey, ultimately leading to functional impairments, emotional distress, and even depressive states [1,2]. The prevalence of hemorrhoids increases as the pregnancy progresses due to the increased pressure on the pelvic veins and the strain on the rectal veins during delivery. Increased progesterone and estrogen hormones during pregnancy can weaken the veins and lead to hemorrhoids. Other risk factors, such as constipation (a predominant symptom during pregnancy), obesity, and a sedentary lifestyle, can also contribute to developing hemorrhoids during pregnancy [1,3].

The management of hemorrhoids is initially aimed at reducing the symptoms of hemorrhoids, i.e., bleeding, pain, itching, and anal discomfort. Management strategies for hemorrhoids include initiation with a conservative treatment approach for minor symptoms and minimally non-invasive techniques or surgery for complex symptoms. Conservative treatment strategies include intake of dietary regimen with fiber supplements, sitz bath, use of stool softeners and laxatives, local anesthetics, astringents, vasoconstrictors, corticosteroids, analgesics, and antipruritic drugs. Currently, the use of oral and topical flavonoid treatments is considered the most effective conservative treatment approach for the management of patients with hemorrhoids [1,4]. The Association of Colon and Rectal Surgeons of India (ACRSI) practice guidelines recommend the management of hemorrhoids during pregnancy using a conservative approach, including diet and lifestyle modifications (intake of fiber-rich diet, high liquid intake, constipation cessation, personal cleanliness, and lying on the left side to relieve pain and other symptoms) and medical therapy. Medical therapy with flavonoids' class of drugs (micronized purified flavonoid fraction (MPFF)) is safe and effective in the management of hemorrhoids in pregnant women (except in the first trimester), and in the antenatal period, maintenance treatment significantly reduces the frequency and duration of relapses of symptoms of acute hemorrhoids [5,6]. Among the non-micronized oral flavonoids available (diosmin, troxerutin, and hydroxyethylrutosides), only hydroxyethylrutosides were studied in pregnant women. They were found to be safe and effective in the treatment of hemorrhoids in pregnant women [7-9].

Limitations of the available pharmacological and surgical treatment options for hemorrhoids include risk of recurrence of symptoms, unable to target multiple pathological factors, and development of complications, such as post-procedural pain and discomfort, anal stenosis, hemorrhage, incontinence, and residual piles. Therefore, there is a need of pharmacological agents that provides quick and long-term relief from hemorrhoidal symptoms by targeting multiple pathological processes in the development of hemorrhoids [10,11]. 

*Euphorbia prostrata *dry extract is known as the “first in the world innovations” launched in India in the year 2008 after receiving approval from the Drug Controller General of India (DCGI). *Euphorbia prostrata* has obtained patents in India, United States, and European regulated markets, allowing its use as a treatment for anorectal diseases, such as hemorrhoids and colonic diseases. It is available in the form of 100 mg tablet and cream 1% for the treatment of Grade 1 and 2 hemorrhoids [10,12]. It is also recommended for providing relief from symptoms in patients with Grade 3 and 4 hemorrhoids who have undergone a hemorrhoidectomy. In addition, it is offered in the form of a fixed-dose combination with 500 mg of calcium dobesilate for oral administration and a topical combination with 3% w/w lidocaine [10]. The active components identified in *Euphorbia prostrata* dry extract are flavonoids, phenolic compounds, and tannins. Flavonoids (apigenin, apigenin-7 glucoside, luteolin, and luteolin-7 glucoside) and phenolic acid (ellagic and gallic acids) have been reported to have anti-inflammatory, analgesic, antioxidant, hemostatic, antithrombotic, wound healing, and vasoprotective actions. Tannins are known to possess astringent and hemostatic properties. Furthermore, preclinical studies on the extract have confirmed its wound healing and anti-hemorrhoidal activity. Importantly, preclinical studies on safety using the standardized extract of *Euphorbia prostrata* have demonstrated that it does not affect the cardiovascular, respiratory, central nervous, or gastrointestinal systems [10].

*Euphorbia prostrata* is available as a prescription drug since last one and half decade with established clinical efficacy and safety in patients with hemorrhoids. However, till date, there is no published literature evaluating its use in pregnant women with hemorrhoids. Therefore, in order to explore its use in the special population involving pregnant women with hemorrhoids, the present study "Management of Hemorrhoids during Pregnancy with EuphoRbia prostrata (tablet 100 mg plus cream 1% - A singlE arm prospective muLti-centrIc Efficacy and saFety study: Hem-Preg-RELIEF" aimed to assess the efficacy and safety of Sitcom® tablet and Sitcom® cream in the hemorrhoids during pregnancy.

## Materials and methods

Study design

This was a non-randomized, open-label, prospective single-arm interventional multicentric study conducted between 2021 and 2022 at three different centers situated in Pune, India (Healing Hands Clinic (fourth floor, Millenium Star Extension, Dhole Patil Road), Healing Hands Clinic (first floor, Crystal Empire Building, Baner Road), and Healing Hands Clinic (second floor, Premier Plaza, Chinchwad).

Ethical considerations

The study was performed in accordance with the principles stated in the Declaration of Helsinki. Prior to study initiation, approval of the study protocol was obtained from the Healing Hands Clinic Independent Ethics Committee (IEC) (Reg. No. ECR/283/Indt/MH/2017/RR-21), based at Millennium Star Extension, Dhole Patil Road, Pune, India. Written informed consent was obtained from each participant before the study enrollment.

Eligibility criteria

Women ≥18 years of age with a complaint of hemorrhoids during pregnancy and who were willing to participate in the study and sign the informed consent form were included in this study.

Women in the first trimester of their pregnancy, having hypersensitive predisposition or hypersensitive to any of the components of the study drug, and with a clinically significant comorbid condition that, in the investigator's opinion, could affect patients were excluded from this study.

Study drug

The study drug was Sitcom (*Euphorbia prostrata*) tablet (oral) (manufactured by Panacea Biotec Ltd., India) 100 mg tablet once daily for 14 days on empty stomach and Sitcom (*Euphorbia prostrata*) cream 1% w/w (manufactured by Panacea Biotec Ltd., India) to be applied locally at least twice daily and after each act of defecation for 14 days.

Study procedure

**Figure 1:**
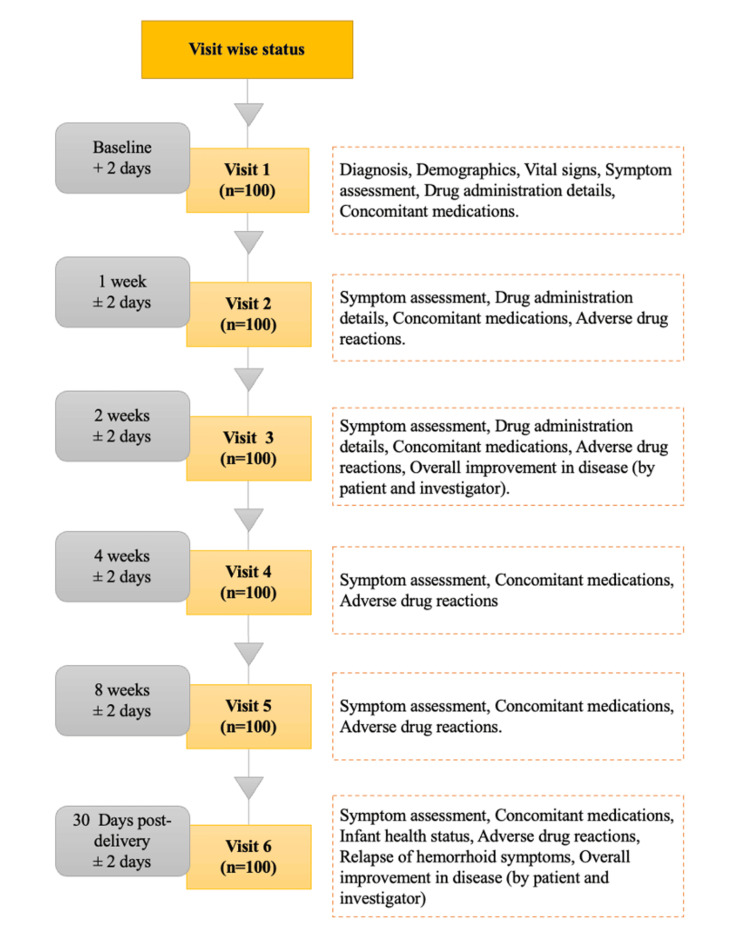
Summary of assessments across study visits

At the time of screening and enrollment (visit 1), medical history was noted from eligible women with a complaint of hemorrhoids during pregnancy. A physical examination was done to record height, weight, and vital signs (blood pressure and pulse rate). Symptom assessment in each patient includes cessation of per-rectal bleeding, pain at defecation, itching, exudation, and swelling. The enrolled patients were given *Euphorbia prostrata* 100 mg tablets and *Euphorbia prostrata* 1% w/w cream and advised to take the tablet at the recommended dose along with the local application of cream as a combination therapy for 14 days. Patients were advised to come for follow-up visits at the end of one week (visit 2), two weeks (visit 3), four weeks (visit 4), eight weeks (visit 5), and 30 days post-delivery (visit 6). The protocol-specified visits are summarized in a study flowchart (Figure 1).

At each visit, the investigator conducted symptom assessment and recorded drug administration details, concomitant medications, drug safety details and adverse events (AEs), and serious adverse events (SAEs), if any reported by the patients. The investigator was assigned to enter the patient’s data in the respective case report form (CRF). All adverse events observed or reported/volunteered by patients were recorded in the CRF with information about severity (mild: usually transient in nature and generally not interfering with normal activities; moderate: sufficiently discomforting to interfere with normal activities; severe: prevents normal activities) and possible relation to the study medication. In addition, at the end of two weeks and at 30 days postpartum follow-up, the physician and the patient assessed the overall improvement in the disease.

The laboratory tests and other special investigations deemed necessary in the investigator's opinion, for the patient's safety were undertaken during the study. If a patient discontinues from the study medication on or before visit 2 (either drop out or due to any adverse drug reaction), visit 2 was considered the study completion visit and activities listed for a visit 3 were not performed. Patients were instructed to report at the study site for any AE and SAE observed during the study.

Endpoints

Efficacy endpoints were scored as 0 for no symptoms, 1 for mild symptoms, 2 for moderate symptoms, and 3 for severe symptoms. Efficacy endpoints were to assess the relief from per-rectal bleeding, pain and itching at defecation, exudation, and swelling from baseline to end of the study, the proportion of patients having a relapse of symptoms and requiring re-treatment initiation during the study duration. The patient’s and physician’s assessment of efficacy was done on the overall improvement in the disease condition from baseline to end of week 2 and from baseline to end of study (30 days post-delivery). Profile of infant health status was carefully assessed (in terms of general well-being, breastfeeding status, vitals, abnormality, and relationship with the study drug). The safety endpoint was to evaluate the number of adverse events during the study duration.

Statistical analysis

Data analysis was done using IBM SPSS Statistics for Windows, version 10 (SPSS Inc., Chicago, USA). Descriptive data are expressed as mean and standard deviation (SD) and numbers (No.) and percentages (%). Comparative analysis between qualitative variables was done using a chi-square test, and quantitative parameters were evaluated using the Wilcoxon signed-rank test. A p‑value <0.05 was considered statistically significant.

## Results

A total of 100 patients were included in this study. The mean (SD) age was 24.1 (3.8) years. The mean weight and height of the patients were 61.2 (12.2) kg and 5.3 (0.3) ft, respectively. The proportion of patients in the second trimester and third trimester was 58.6% and 41.1%, respectively. The majority (75.0%) of the patients had internal hemorrhoids, and 25.0% of patients had a combination of internal and external hemorrhoids. In total, 15.0% of the patients were on laxatives. The demographic details are summarized in Table 1.

**Table 1:** Demographic characteristics of the patients Data presented as n (%), unless otherwise specified. N = 100, unless otherwise specified.

Parameter	Number of patients (n = 100)
Age (years), mean (SD)	24.1 (3.8)
Weight (kg), mean (SD)	61.2 (12.2)
Height (ft), mean (SD)	5.3 (0.3)
Systolic blood pressure (mm Hg), mean (SD)	117.4 (7.8)
Diastolic blood pressure (mm Hg), mean (SD)	74.9 (8.5)
Pulse rate (per min), mean (SD)	79.4 (8.9)
Pregnancy trimester	(n=99)
Second	58 (58.6)
Third	41 (41.4)
Type of hemorrhoids	
Internal	75 (75.0)
Internal and external	25 (25.0)
Administration of laxatives	15 (15.0)
Treatment type	
Combination of *Euphorbia prostrata* 100 mg tablets plus *Euphorbia prostrata* cream 1% w/w till 2 weeks	100 (100.0)
Additional *Euphorbia prostrata* 100 mg tablets (till 4 weeks)	13 (13.0)
Additional *Euphorbia prostrata* cream (till 4 weeks)	15 (15.0)

Symptoms and treatment profile

**Figure 2:**
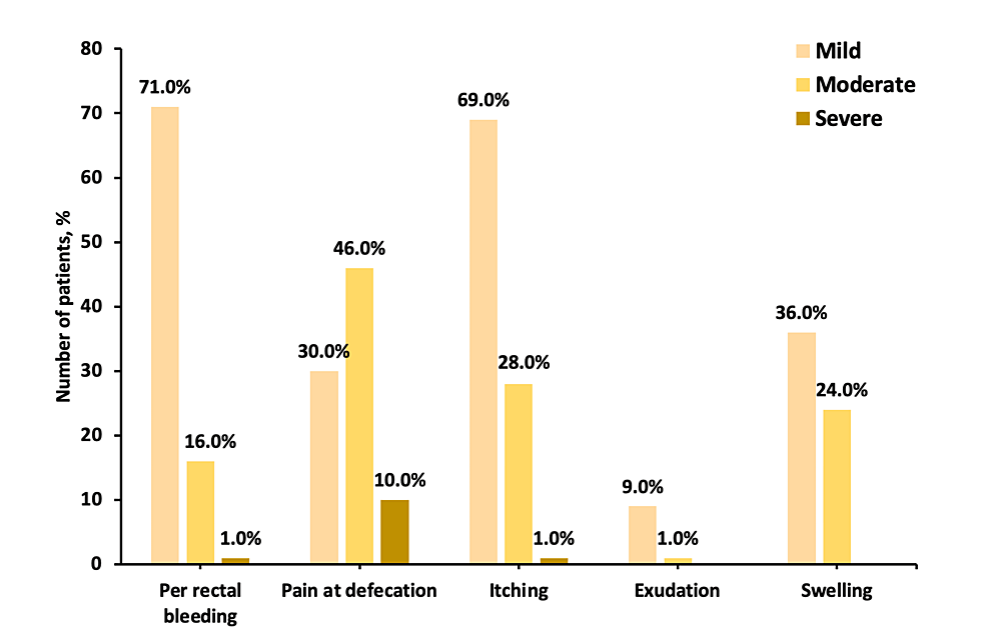
Severity grading of symptoms in patients at baseline Data presented as %.

All the patients received a combination therapy of *Euphorbia prostrata* tablet and cream for two weeks. Furthermore, 13 patients were administered *Euphorbia prostrata* tablet, and 15 patients were given *Euphorbia prostrata* cream during the next two more weeks of the study period based on the individual patient’s need (Table 1). The patients were not treated with any other concomitant medicines for hemorrhoids, which may influence the outcomes of the study drug, except laxatives, which were allowed as per the requirement of individual patients for underlying constipation problem. The majority of patients had mild (71.0%) per-rectal bleeding, followed by 16.0% having moderate per-rectal bleeding. The majority of patients had moderate (46%) pain at defecation, followed by 30.0% of patients with mild pain and 10% with severe pain at defecation. A total of 69.0% of patients had mild itching, 28.0% had moderate itching, and 1.0% had severe itching. Exudation and swelling were of mild to moderate severity (Figure 2).

Efficacy endpoints

**Figure 3:**
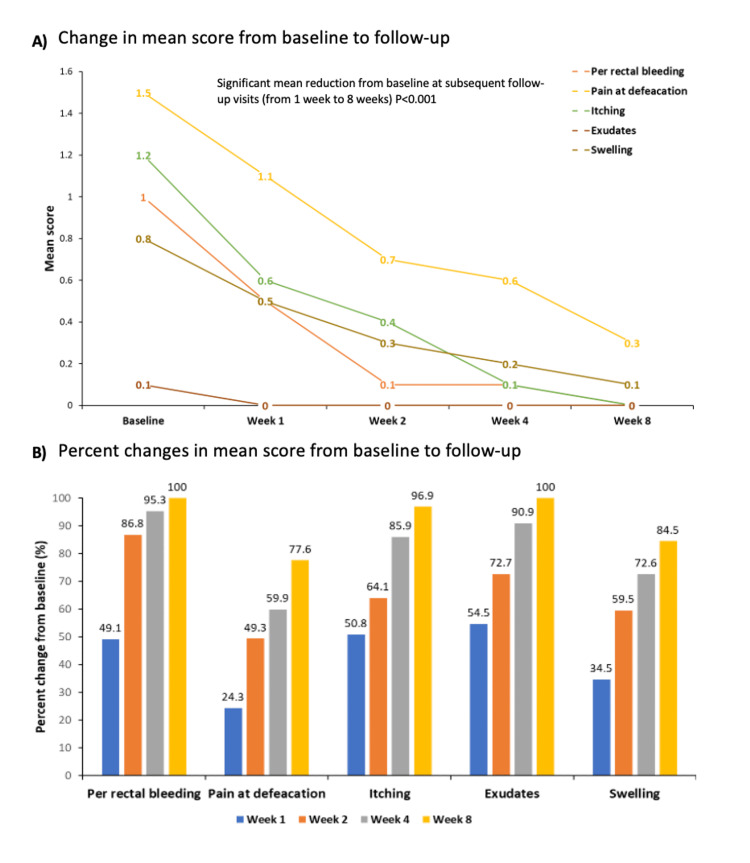
A) Changes in hemorrhoidal symptoms, B) proportion of changes in the mean score from baseline to follow-up of eight weeks For Fig. A, data are presented as mean, and for Fig. B, data are presented as %.

There was a significant mean reduction from baseline at subsequent follow-up visits (from one week to eight weeks follow-up) in different symptoms, such as per-rectal bleeding, pain during defecation, itching, exudation, and swelling (p < 0.001) (Figure 3A and Table 2).

**Table 2:** Changes in hemorrhoidal symptoms from baseline to follow-up of eight weeks Data presented as mean (SD), unless otherwise specified.

Duration	Baseline	1 week	2 weeks	4 weeks	8 weeks	p-value
Per rectal bleeding						
Mean score	1.0 (0.5)	0.5 (0.5)	0.1 (0.3)	0.1 (0.2)	0.0	<0.001
Mean change from baseline	-	-0.5 (0.5)	-0.9 (0.5)	-1.0 (0.5)	-1.0 (0.5)	<0.001
Mean score (%)	-	49.1	86.8	95.3	100.0	-
Pain at defecation						
Mean score	1.5 (0.8)	1.1 (0.6)	0.7 (0.4)	0.6 (0.4)	0.3 (0.4)	<0.001
Mean change from baseline	-	-0.3 (0.4)	-0.7 (0.6)	-0.9 (0.7)	-1.1 (0.8)	<0.001
Mean score (%)	-	24.3	49.3	59.9	77.6	-
Itching						
Mean score	1.2 (0.5)	0.6 (0.5)	0.4 (0.5)	0.1 (0.3)	0.0 (0.2)	<0.001
Mean change from baseline	-	-0.6 (0.4)	-0.8 (0.5)	-1.1 (0.4)	-1.2 (0.5)	<0.001
Mean score (%)	-	50.8	64.1	85.9	96.9	-
Exudates						
Mean score	0.11 (0.3)	0.05 (0.2)	0.03 (0.1)	0.01 (0.1)	0.0	<0.001
Mean change from baseline	-	-0.06 (0.2)	-0.08 (0.2)	-0.10 (0.3)	-0.11 (0.3)	<0.001
Mean score (%)	-	54.5	72.7	90.9	100.0	-
Swelling						
Mean score	0.8 (0.7)	0.5 (0.5)	0.3 (0.4)	0.2 (0.4)	0.1 (0.3)	<0.001
Mean change from baseline	-	-0.2 (0.4)	-0.5 (0.6)	-0.6 (0.6)	-0.7 (0.7)	<0.001
Mean score (%)	-	34.5	59.5	72.6	84.5	-

After one week of treatment, the mean score of per-rectal bleeding showed a significant reduction of 49.1% from baseline, which further reached a significant reduction of 86.8% at the end of week 2, 95.3% at the end of week 4, and 100% at the end of week 8. The mean pain score at defecation after one week of treatment showed a significant reduction of 24.3% from baseline, which further dropped to 49.3% at week 2, 59.9% at week 4, and 77.6% at the end of week 8. After one week of treatment, the mean score of itching showed a significant fall of 50.8% from baseline, which further reached a significant reduction of 64.1% at the end of week 2, 85.9% at the end of week 4, and 96.9% at the end of week 8. Similarly, the mean score of exudations showed a significant drop of 54.5% after one week from baseline, 72.7% at two weeks, and 90.9% at four weeks, further dropping to 100.0% at the end of eight weeks of treatment from baseline. These trends were consistent in the mean score of swelling as well, which showed a reduction of 34.5% after one week of treatment from baseline, 59.5% after two weeks, 72.6% after four weeks, and 84.5% after eight weeks of treatment from baseline (Figure 3B).

Profile of overall improvement in the disease

**Figure 4:**
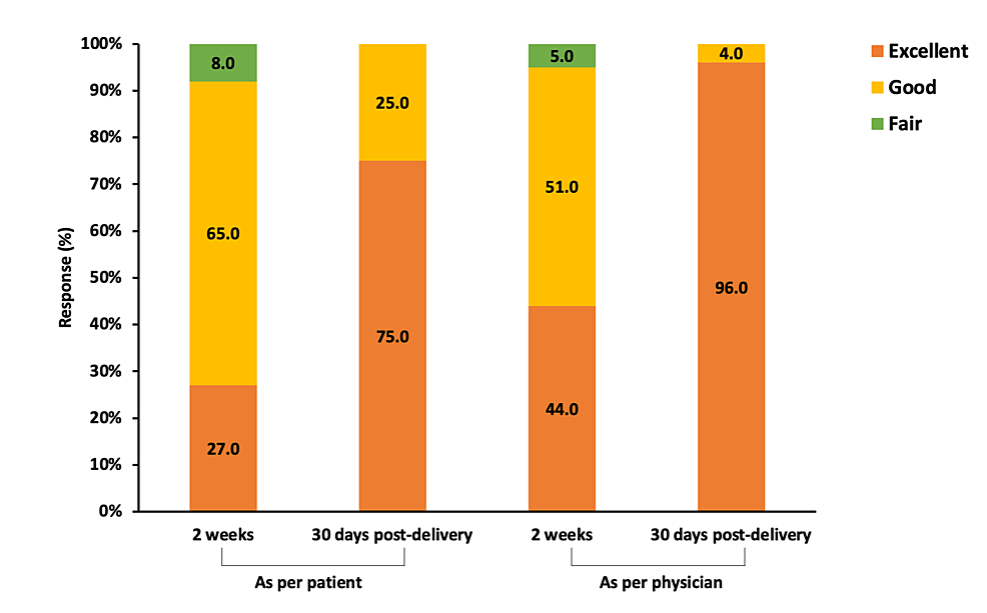
Profile of overall improvement in disease Data presented as %.

After two weeks of treatment, as per the patients’ evaluation, 92.0% of the patients showed good to excellent improvement in the disease. As per the physician’s evaluation, 95.0% of the patients showed good to excellent improvement in the disease. After 30 days post-delivery, 100.0% of the patients showed good to excellent improvement in the disease (Figure 4).

Profile of the infant health status

The profile of the infant health status assessment among the study patients showed that the general well-being of all infants was healthy (100%), breastfeeding was normal (100%), and all vitals were within normal limits (100%). There were no anomalies found and no relationship of the study drug with infants’ health status observed.

Relapse of hemorrhoidal symptoms

A relapse in hemorrhoidal symptoms was observed in 12 patients at the end of the study (after 30 days post-delivery follow-up). Among these, nine patients showed relapse of symptoms with the intensity of <10% and three patients with the intensity of <25% as compared to their baseline symptoms. All these patients with relapse in hemorrhoidal symptoms however did not require any re-treatment (Table 3).

**Table 3:** Relapse of hemorrhoidal symptoms Data presented as n (%).

Parameter	Number of patients (n=100)
Relapse of hemorrhoidal symptoms	12 (12.0)
Symptoms same as initiation of study	-
Relapse of symptoms	(n=12)
<75% intensity	0 (0)
<50% intensity	0 (0)
<25% intensity	3 (25.0)
<10% intensity	9 (75.0)
Re-treatment required	None

Safety endpoints

There were no reports of any adverse events in the patients throughout the study period.

## Discussion

Gastrointestinal disorders are common during pregnancy; approximately 0.2-1% of pregnant women need to visit a general surgeon during their pregnancies [13]. Hemorrhoidal disease is a common anorectal disease observed among women during pregnancy. It is one of the frequently encountered ailments in the general population in routine clinical settings across India [5,14]. Treatment with oral and topical flavonoid agents effectively helps manage patients with hemorrhoids [1,15].

In 2008, the *Euphorbia prostrata* dry extract, containing mainly flavonoids, phenolic acid, and tannins, was approved by DGCI as a “drug of modern medicine” in the form of oral tablet and topical cream for the management of hemorrhoids [16]. With a unique multimodal mechanism of action, *Euphorbia prostrata* helps in controlling inflammation and preventing the capillary bleeding, and on the basis of beneficial observations from previous preclinical and clinical studies that demonstrated its efficacy and safety in grade I and grade II symptomatic hemorrhoids, it is approved for the use in the management of patients with hemorrhoids. However, to date, no study has evaluated efficacy and safety of this drug in pregnant women. This is the first study to establish the efficacy and safety profile of the study drug in a special population of pregnant women with a complaint of hemorrhoids. An overall observation of the present study that involved a total of 100 pregnant women showed efficacy and tolerability of *Euphorbia prostrata *100 mg tablet and cream 1% w/w in ameliorating hemorrhoidal symptoms after treatment for two weeks. 

In the present study, the combination therapy of *Euphorbia prostrata* 100 mg tablet and cream for two weeks effectively improved hemorrhoidal symptoms. Most hemorrhoidal symptoms were mild to moderate, whereas 10% of patients reported severe pain during defecation. Furthermore, a gradual improvement in relief from per-rectal bleeding and exudation was observed at each follow-up visit from two weeks (86.8% and 72.7%) to four weeks (95.3% and 90.9%) to eight weeks (100.0% each). Complete relief from per-rectal bleeding and exudation was seen at the end of eight weeks, whereas other hemorrhoidal symptoms, such as pain at defecation (77.6%), itching (96.9%), and swelling (84.5%), were also improved to an acceptable extent at the end of eight weeks of treatment, suggesting that the symptoms were almost cured with the given treatment in these patients. These observations indicate that combination therapy used in the study for two weeks had long-term efficacy that continued till eight weeks. This overall trend of improvement in the hemorrhoidal symptoms was consistent with the previous studies evaluating the efficacy of *Euphorbia prostrata* dry extract tablet and cream in different patient populations [16-18].

In accordance with the present study findings, a pilot study evaluating the efficacy and safety of 100 mg of *Euphorbia prostrata *in a patient population (n = 120) with early-grade symptomatic hemorrhoids demonstrated that the study drug was efficacious in achieving hemorrhoidal symptom relief in more than 80% of patients at the end of 14 days. None of the patients reported any adverse events or recurrence of symptoms at the end of the three-month follow-up [17]. An observational study with more than 1,850 patients reported that the maximum improvement in hemorrhoidal symptoms was seen in the first three days of the treatment with *Euphorbia prostrata* dry extract, indicating a rapid recovery from hemorrhoidal symptoms during the early course of the treatment [18]. A similar single-arm interventional study from West Bengal, India, involving 30 patients with hemorrhoids, evaluated the efficacy of an oral fixed drug combination (FDC) of *Euphorbia prostrata* 100 mg plus calcium dobesilate 500 mg and topical FDC of *Euphorbia prostrata* extract 1% w/w plus lidocaine 3% w/w. The optimal symptom relief was seen during the first four days of the treatment. Symptoms, such as bleeding, pain, and swelling, gradually cured (91.66%, each) after two weeks of treatment, while itching and exudation completely disappeared (100%, each). These observations emphasized the importance of a multimodal targeted approach for managing patients with hemorrhoids [16].

In our study, none of the patients reported any adverse events. Relapse of minor symptoms occurred in a small proportion of patients who did not require further treatment. These safety observations concord with the available study in the literature [17]. In addition, the present study revealed that the study treatment did not affect infant health status. Therefore, all these observations indicate that the study drug treatment was well tolerated in women during pregnancy and did not impact the infant health.

In addition, this study assessed overall improvement in disease conditions from the patients' and physicians’ perspectives. We demonstrated that the two weeks of treatment improved disease conditions in more than 91% of the patients. At the end of the 30 days postpartum, all patients showed good to excellent improvement in a disease condition.

It is well established that *Euphorbia prostrata* is associated with a wide profile of beneficial properties, such as anti-inflammatory, antioxidant, wound healing, anti-allergic, pain relieving, venotonic, and anti-edema activities [10]. These properties contribute to the rapid and consistent improvement in hemorrhoidal symptoms in patients with hemorrhoids [10]. Besides its approved use for early-grade hemorrhoids, in 2009, the DCGI also sanctioned the oral formulation of *Euphorbia prostrata* dry extract (100 mg taken twice daily for 14 days) for the treatment of advanced hemorrhoids (specifically, grades 3 and 4) following hemorrhoidectomy [10].

Although this is a first study assessing the efficacy and safety of *Euphorbia prostrata* dry extract in pregnant women, it has its own limitations. Currently, there is no literature available with regard to the use of *Euphorbia prostrata* in hemorrhoid treatment during pregnancy. Although the sample size is small, this pivotal study adds valuable evidence and forms the basis for further, larger studies. Our study will serve as a foundation for future studies with a large sample size will explore and provide insights in the use of *Euphorbia prostrata *in the management of hemorrhoids during pregnancy.

## Conclusions

The anti-inflammatory, analgesic, antioxidant, hemostatic, wound healing, venotonic, and anti-hemorrhoidal properties of *Euphorbia prostrata* make it a potential pharmacological agent that may act on the multiple pathophysiological pathways involved in the development of hemorrhoids with a unique multimodal mechanism of action and can provide accelerated wound healing, prevent capillary bleeding, and control inflammation. Overall observations of this study support the efficacy and safety of using 100 mg of *Euphorbia prostrata* tablet and cream 1% to improve hemorrhoidal symptoms in pregnant women with hemorrhoids. The substantial improvement in disease condition, no requirement of re-treatment, and no impact on infant health status suggest that the combination therapy with *Euphorbia prostrata* dry extract 100 mg tablet and cream may be considered a promising option for the management of hemorrhoids in women during pregnancy.
